# Ventilation Strategies during Neonatal Cardiopulmonary Resuscitation

**DOI:** 10.3389/fped.2018.00018

**Published:** 2018-02-12

**Authors:** Nariae Baik, Megan O’Reilly, Caroline Fray, Sylvia van Os, Po-Yin Cheung, Georg M. Schmölzer

**Affiliations:** ^1^Department of Pediatrics, Medical University Graz, Graz, Austria; ^2^Centre for the Studies of Asphyxia and Resuscitation, Neonatal Research Unit, Royal Alexandra Hospital, Edmonton, AB, Canada; ^3^Department of Pediatrics, University of Alberta, Edmonton, AB, Canada

**Keywords:** infants, newborn, delivery room, neonatal resuscitation, chest compression

## Abstract

Approximately, 10–20% of newborns require breathing assistance at birth, which remains the cornerstone of neonatal resuscitation. Fortunately, the need for chest compression (CC) or medications in the delivery room (DR) is rare. About 0.1% of term infants and up to 15% of preterm infants receive these interventions, this will result in approximately one million newborn deaths annually worldwide. In addition, CC or medications (epinephrine) are more frequent in the preterm population (~15%) due to birth asphyxia. A recent study reported that only 6 per 10,000 infants received epinephrine in the DR. Further, the study reported that infants receiving epinephrine during resuscitation had a high incidence of mortality (41%) and short-term neurologic morbidity (57% hypoxic-ischemic encephalopathy and seizures). A recent review of newborns who received prolonged CC and epinephrine but had no signs of life at 10 min following birth noted 83% mortality, with 93% of survivors suffering moderate-to-severe disability. The poor prognosis associated with receiving CC alone or with medications in the DR raises questions as to whether improved cardiopulmonary resuscitation methods specifically tailored to the newborn could improve outcomes.

## Introduction

Chest compression (CC) is an infrequent event (0.08%) in newborns delivered at near-term and term gestation, and happens at higher frequency (~10%) in preterm deliveries (1–5). In addition, outcome studies of deliveries requiring resuscitation or CC have reported high rates of mortality and neurodevelopmental impairment in surviving children (1–5). The poor prognosis associated with resuscitation requiring CC alone and/or medications in the delivery room (DR) raises questions as to whether improved cardiopulmonary resuscitation (CPR) techniques specifically tailored toward the newborn infant could improve outcomes.

## Asphyxia at Birth

**Figure 1:**
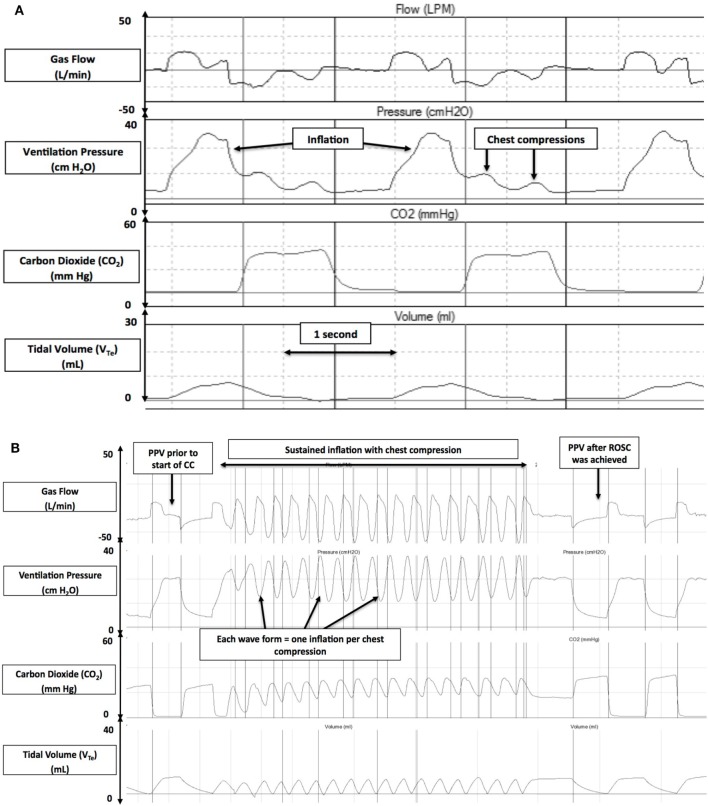
Respiratory waveforms during cardiopulmonary resuscitation in the 3:1 compression:ventilation ratio (3:1 C:V) **(A)** and chest compression (CC) + sustained inflation (SI) **(B)** groups (gas flow, airway pressure, ECO_2_, and tidal volume). Reproduced with permission from Schmölzer et al. (8).

Asphyxia, a condition of impaired gas exchange with simultaneous hypoxia and hypercapnia leading to a mixed metabolic and respiratory acidosis, is the most common reason that newborns fail to make successful transition (6, 7). Asphyxia could result from either failure of placental gas exchange before delivery (e.g., abruption and chorioamnionitis) or deficient pulmonary gas exchange immediately after birth (e.g., apnea, airway obstruction, and respiratory distress syndrome) (6, 7). Asphyxia depresses myocardial function leading to cardiogenic shock, pulmonary hypertension, mesenteric reperfusion, and acute renal failure. Newborn infants present with serve bradycardic or asystole at birth as a consequence of asphyxia. Current resuscitation guidelines recommend to initiate CC if heart rate remains <60/min despite adequate ventilation with supplementary oxygen for 30 s; CC should be then performed at a rate of 90/min with 30 ventilations 3:1 C:V (Figure 1A) (8) to achieve adequate oxygen delivery (9–11).

## Rationale for Using 3:1 Compression to Ventilation Ratio

Neonatal bradycardia or cardiac arrest is caused by hypoxia rather than primary cardiac compromise; therefore, providing ventilation is more beneficial (9–11). However, the optimal C:V ratio that should be used during neonatal resuscitation to optimize coronary and cerebral perfusion while providing adequate ventilation of an asphyxiated newborn remains unknown.

Animal studies on cardiac arrest induced by asphyxia in newborn piglets demonstrated that combining CC with ventilations improves ROSC and neurological outcome at 24 h compared to ventilations or CC alone (12–14). Solevåg et al. performed a study investigating alternating nine CC and three ventilations in asphyxiated piglets with cardiac arrest with the hypothesis that nine CC would generate higher diastolic blood pressure (15). The time to ROSC was similar between the two approaches (150 and 148 s for 3:1 and 9:3 C:V, respectively). Similarly, C:V ratios of 3:1 and 15:2 were compared using the same model (16). Although the 15:2 C:V ratio provided higher mean CC per minute (75 versus 58 for 3:1), time to ROSC was similar between groups (median time of 195 and 150 s for 15:2 and 3:1, respectively) (16). These studies suggest that during neonatal CPR, higher C:V ratios do not improve outcomes, and potentially a higher ventilation rate is needed.

**Figure 2:**
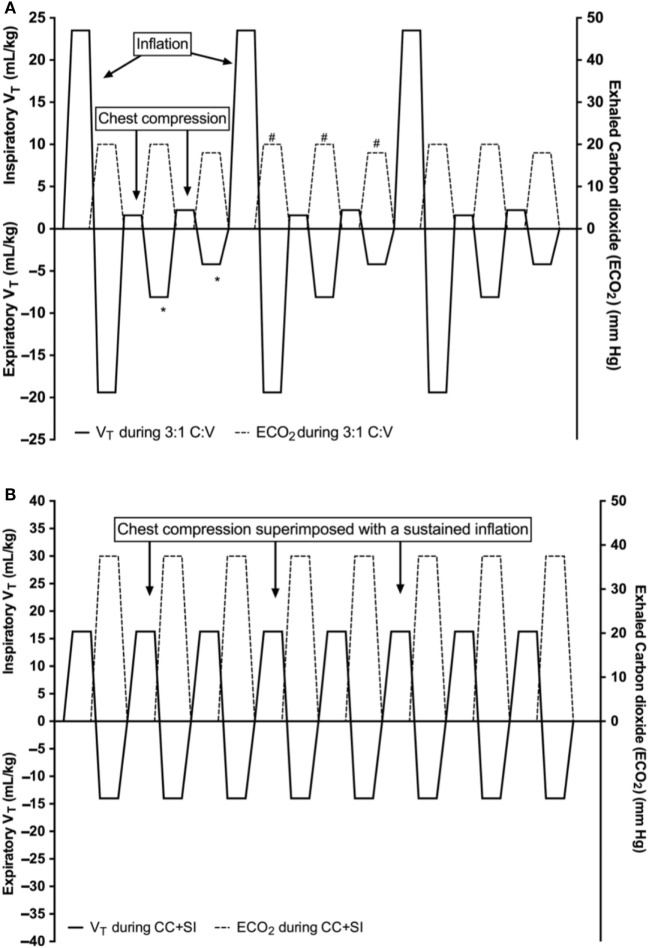
Tidal volume (mL/kg) changes during 3:1 compression:ventilation ratio (3:1 C:V) **(A)** and continuous chest compressions (CCs) superimposed by sustained inflations (SIs) (CC + SI) **(B)**. Reproduced with permission from Li et al. (23).

This is further supported by manikin studies showing higher ventilation rates during simulated CPR using 3:1 C:V compared with higher C:V ratios (17–19). A more recent neonatal manikin study examined respiratory parameters during neonatal CPR and reported that a 3:1 C:V ratio delivered significantly higher minute ventilation of 191 mL/kg compared to the minute ventilation at 9:3 and 15:2 C:V ratios (140 and 77 mL/kg/min, respectively) (20). A further manikin study compared 3:1 C:V with continuous CC with asynchronous ventilations (CCaV) using 90 CC and 30 non-synchronized inflations and reported significantly higher minute ventilation in the CCaV group compared to the 3:1 group (221 versus 191 mL/kg/min, respectively) (21). Schmölzer et al. compared 3:1 C:V CPR with CCaV in a piglet model of neonatal asphyxia and reported similar minute ventilation (387 versus 275 mL/kg) (22). There was also a similar time to ROSC (143 and 114 s for 3:1 and CCaV, respectively) and survival (3/8 and 6/8, respectively). The same manikin (21) and animal study (22) also reported similar tidal volume (*V*_T_) delivery between 3:1 C:V and CCaV [manikin study *V*_T_ 6.4 and 5.6 mL/kg (21), respectively and animal study *V*_T_ 14.7 versus 11.0 mL/kg (22)]. In a secondary analysis of the study by Schmölzer et al. (22), Li et al. reported that during 3:1 C:V a cumulated loss of *V*_T_ of 4.5 mL/kg occurs for each 3:1 C:V cycle (Figure 2A) (23). Similarly, during CCaV, a cumulated loss of *V*_T_ of 9.1 mL/kg for each cycle of three CC and one inflation were observed (23). This suggests a potential loss in *V*_T_ during CC, which could cause lung derecruitment, hence hamper oxygenation and therefore ROSC. A recent pilot randomized trial in the DR reported that the exhaled CO_2_ was significantly higher in the CC + sustained inflation (SI) group with 11 (9) mmHg compared to 2 (1) mmHg (*p* < 0.001) in the 3:1 C:V ratio group during CPR suggesting improved gas exchange in the CC + SI group (24). Further, an argument of synchronized CPR is the potential interference of non-synchronized CC with *V*_T_ delivery, resulting in impairment of oxygen delivery. However, the study by Schmölzer et al. observed that 29 and 25% of manual inflations were similarly affected during CC using CCaV or 3:1 C:V CPR (22), respectively. These studies suggest that no advantages (e.g., oxygen delivery and *V*_T_ or minute ventilation) of using CCaV compared to 3:1 C:V.

## Rational for Using Continuous CCs with SI

Reoxgenation and adequate blood flow are the cornerstones of neonatal CPR. Any effective resuscitative maneuver should increase blood flow and optimize oxygen delivery. In addition to standard CPR, maneuvers that raise intrathoracic pressure can significantly increase carotid blood flow during CPR. Chandra et al. provided ventilation at a high airway pressure while simultaneously performing CC in an animal model and demonstrated increased carotid flow without compromising oxygenation (25). Further, studies in preterm lambs have demonstrated that an SI also increases intrathoracic pressure without impeding blood flow (26). In the resuscitation of asphyxiated newborn piglets, Schmölzer et al. recently reported that passive ventilation during CC, achieved by superimposing CC with an SI (CC + SI) (Figure 1B) (8), significantly improved hemodynamics, minute ventilation, and time to ROSC compared to the current approach of using a 3:1 C:V ratio (mean arterial pressure: 51 versus 31 mmHg; pulmonary arterial pressure: 41 versus 31 mmHg; mean minute ventilation: 936 versus 623 mL/kg; and median time to ROSC: 38 versus 143 s, respectively) (8). However, the study by Schmölzer et al. used a CC rate of 120/min (in the CC + SI group), which is higher than the currently recommended CC rate of 90/min, which could have also added to the improved outcomes. A recent study using a perinatal cardiac arrest lamb model with transitioning fetal circulation and fluid filled lungs reported that CC + SI is as effective as 3:1 C:V ratio in achieving ROSC (27).

### Rate of CC

A recent mathematical model suggests that CC rates higher than the currently recommended 90 CC/min could optimize systemic perfusion (28). This model further suggests that the most effective CC rate depends on body size and weight, which would translate to 180 CC/min for term infants and even higher rates for preterm infants (28). However, a recent simulation study comparing various CC rates (e.g., 90 versus 120 CC/min) reported faster fatigue with increasing CC rates (29). Overall, using CC rates of 90 CC/min was the least fatiguing and the most preferred method of neonatal CPR compared to that using 120 CC/min (29). This is further supported by a study by Solevåg et al. who quantified the force used during CC and reported fatigue occurring faster with increasing CC rates (30). These studies suggest that a rate of 90 CC/min would be the least fatiguing rate. Further, a recent randomized animal trial compared CC + SI using CC rates of 90 versus 120 CC/min and reported similar time of ROSC, survival rates, and respiratory parameters during CPR (31). During CC, carotid blood flow, mean arterial pressure, and percentage change in ejection fraction and cardiac output were higher in the CC + SI 90/min group compared to CC + SI 120/min (31). This further supports that higher CC rates do not improve systemic perfusion and that the current recommendation of 90 CC/min are sufficient to achieve systemic perfusion. This is further supported by a randomized animal trial comparing CC + SI using a rate of 90/min with 3:1 C:V. CC + SI significantly reduced the median (IQR) time of ROSC, i.e., 34 s (28–156 s) versus 210 s (72–300 s) in the 3:1 group (*p* = 0.048). CC + SI also significantly reduced the requirement for 100% oxygen, improved respiratory parameters, and resulted in a similar hemodynamic recovery (32). Furthermore, the study reported no significant differences in the concentrations of interleukin (IL) IL-1β, IL-6, IL-8, or tumor necrosis factor in lung tissue homogenates (32). This suggests that SI does not increase the risk of brain injury as recently suggested in two meta-analyses (33, 34).

### Adequate Ventilation during CC

Providing adequate ventilation to achieve reoxgenation is a cornerstone of neonatal CPR. Current best practice is to provide 90 CC and 30 ventilations that are coordinated during a pause (9). The purpose of inflations during CC is to deliver an adequate *V*_T_ to facilitate gas exchange. However, delivery of an adequate *V*_T_ during CPR remains difficult. Several DR studies reported that mask ventilation is the most difficult task during neonatal CPR. *V*_T_ delivery could be compromised due to mask leak or airway obstruction (35–43), causing inadequate oxygen delivery to any asphyxiated newborn. A recent case by Li et al. reported a large mask leak during mask ventilation in the DR, which resulted in severe bradycardia and the need for neonatal CC (44). In addition, once CC was started, mask leak further increased (44). This is further supported by manikin studies, which reported decreased expiratory *V*_T_ once CC were started (20, 45, 46). Binder-Heschl et al. examined human or monitor feedback during simulated neonatal CPR using a leak-free manikin and reported lower expiratory *V*_T_ in all groups compared with mask ventilation alone (46). These studies suggest a decrease in expiratory *V*_T_ once CCs are initiated. A loss in expiratory *V*_T_ could cause lung derecruitment, which could hamper oxygenation and therefore ROSC. This has been recently confirmed in an animal model of neonatal CPR. In a secondary analysis of the study by Schmölzer et al. (22), Li et al. reported that during a significant loss of expiratory *V*_T_ compared to inspiratory *V*_T_ over each 3:1 C:V cycle (mean total inspiratory *V*_T_ was 27.2 mL/kg and mean total expiratory *V*_T_ 31.7 mL/kg = a loss of 4.5 mL/kg per 3:1 C:V cycle) (Figure 2A) (8, 23). In contrast, no *V*_T_ loss was observed in CC + SI. Instead, continuous lung recruitment and establishment of functional residual capacity were observed (mean total inspired *V*_T_ was 16.3 mL/kg and mean total expiratory was 14 mL/kg = a gain of 2.3 mL/kg/CC + SI cycle) (Figure 2B) (8, 23). This improvement in *V*_T_ delivery might lead to better alveolar oxygen delivery and lung aeration. More importantly, the initial study by Schmölzer et al. (22) and secondary analysis by Li et al. (23) describe passive *V*_T_ delivery during each CC cycle. Similar observations have been reported by Tsui et al. (47) in children under the age of 17 years undergoing any surgery requiring general anesthesia and endotracheal intubation. Tsui et al. compared *V*_T_ after induction of general anesthesia before and after intubation by applying a downward force on the chest, which was not greater than the patient’s weight in seven infants (47). Overall, median (IQR) *V*_T_ generated before and after intubation was 2.4 (0.8–4.0) and 2.0 (0.4–3.6) mL/kg, respectively (47). Although, Tsui et al. only applied gentle chest pressure, they could achieve ~33% of an infant’s physiological *V*_T_ of 5–7 mL/kg. During CC, the mean applied forced is ~10 kg (3–4 times the weight of the newborn infant) (30), which would result in adequate *V*_T_ delivery during CC + SI. Further, Solevåg et al. determined the distending pressure needed to achieve sufficient *V*_T_ delivery using different models (manikin and cadaver piglets) during CC + SI (48). Distending pressure and *V*_T_ correlated in cadaver piglets (*r* = 0.83, *p* < 0.001), manikin (*r* = 0.98, *p* < 0.001), and combined data (*r* = 0.49, *p* < 0.001). *V*_T_ was delivered during chest recoil during CC in both models. In cadaver piglets, a distending pressure ~25 cmH_2_O was needed to achieve an adequate *V*_T_. This study suggests that chest recoil generates *V*_T_ depending on an adequate distending pressure, and that a pressure of ~25 cmH_2_O will be needed to achieve adequate *V*_T_ delivery. In addition, this has been recently shown in the first randomized controlled trial in the DR by Schmölzer et al. (24). Overall, the mean (SD) time to ROSC was significantly shorter in the CC + SI group with 31 (9) s compared with 138 (72) s in the 3:1 C:V group (*p* = 0.011). Infants in the CC + SI group had significantly higher respiratory rates with 91 (1) versus 29 (2) inflations/min in the 3:1 C:V group (*p* = 0.0001). The delivered tidal volume ranged between 0.6 and 4.4 mL/kg in the CC + SI group and 0.8 and 4.5 mL/kg in the 3:1 C:V group. Median (IQR) minute ventilation was significantly higher in the SI + CC group compared to the 3:1 C:V group 165 (85–216) versus 101 (48–110) mL/kg/min (*p* = 0.0001). This is currently further studied in a multicenter cluster randomized trial “CC + SI versus 3:1 C:V ratio during neonatal CPR (SURV1VE)—NCT02858583” to study this in a larger patient population.

## Quality and Depth of CC and/or the Administration of Epinephrine

Improved left ventricular ejection fraction have been postulated after review of computer tomography images of neonates when CC with a 1/3 anterior–posterior chest diameter was compared with a 1/4 anterior–posterior chest diameter (49). The current neonatal resuscitation guidelines recommend a 1/3 anterior–posterior chest diameter during CPR (9–11). Adequate CC depth is important to optimize cardiac output. However, compressing to deep could cause rib fractures, cardiac contusion, and other thoracic injuries (7). While compressing to shallow might not create the required cardiac output or diastolic blood pressure (50, 51). A retrospective review of six infants (2 weeks to 7.3 months old) with indwelling arterial blood pressure monitoring reported that CC with a 1/2 anterior–posterior chest diameter produced significantly higher systolic blood pressure but similar diastolic blood pressure compared with CC using a 1/3 anterior–posterior chest diameter. However, the optimal CC depth has not been rigorously evaluated in neonates. Furthermore, no study has examined tidal volume delivery with different anterior–posterior chest diameter.

## Conclusion

Successful resuscitation from cardiac arrest or severe bradycardia requires the delivery of high-quality CC while providing adequate ventilation. However, until now, no study has examined different CC techniques during neonatal resuscitation in asphyxiated newborn infants, and randomized controlled trials are urgently needed.

## Author Contributions

Concept, literature search, review of the data, writing of the manuscript, and review of the manuscript: NB, MO, CF, SO, P-YC, and GS.

## Conflict of Interest Statement

The authors declare that the research was conducted in the absence of any commercial or financial relationships that could be construed as a potential conflict of interest.
